# A cross-sectional assessment of the burden of HIV and associated individual- and structural-level characteristics among men who have sex with men in Swaziland

**DOI:** 10.7448/IAS.16.4.18768

**Published:** 2013-12-02

**Authors:** Stefan D Baral, Sosthenes Ketende, Zandile Mnisi, Xolile Mabuza, Ashley Grosso, Bhekie Sithole, Sibusiso Maziya, Deanna L Kerrigan, Jessica L Green, Caitlin E Kennedy, Darrin Adams

**Affiliations:** 1 Key Populations Program, Department of Epidemiology, Center for Public Health and Human Rights, Johns Hopkins Bloomberg School of Public Health, Baltimore, MD, USA; 2 Swaziland National AIDS Program (SNAP), Ministry of Health and Social Welfare, Mbabane, Swaziland; 3 Rock of Hope, Manzini, Swaziland; 4 Department of Health Sciences, University of Stellenbosch, Stellenbosch, South Africa; 5 Department of Health, Behavior and Society, Johns Hopkins Bloomberg School of Public Health, Baltimore, MD, USA; 6 Population Services International, Swaziland; 7 Department of International Health, Johns Hopkins Bloomberg School of Public Health, Baltimore, MD, USA

**Keywords:** public health, men who have sex with men (MSM), Africa, HIV, Swaziland, epidemiology

## Abstract

**Introduction:**

Similar to other Southern African countries, Swaziland has been severely affected by HIV, with over a quarter of its reproductive-age adults estimated to be living with the virus, equating to an estimate of 170,000 people living with HIV. The last several years have witnessed an increase in the understanding of the potential vulnerabilities among men who have sex with men (MSM) in neighbouring countries with similarly widespread HIV epidemics. To date, there are no data characterizing the burden of HIV and the HIV prevention, treatment and care needs of MSM in Swaziland.

**Methods:**

In 2011, 324 men who reported sex with another man in the last 12 months were accrued using respondent-driven sampling (RDS). Participants completed HIV testing using Swazi national guidelines as well as structured survey instruments administered by trained staff, including modules on demographics, individual-level behavioural and biological risk factors, social and structural characteristics and uptake of HIV services. Population and individual weights were computed separately for each variable with a data-smoothing algorithm. The weights were used to estimate RDS-adjusted univariate estimates with 95% bootstrapped confidence intervals (BCIs). Crude and RDS-adjusted bivariate and multivariate analyses were completed with HIV as the dependent variable.

**Results:**

Overall, HIV prevalence was 17.6% (*n=*50/284), although it was strongly correlated with age in bivariate- [odds ratio (OR) 1.2, 95% BCI 1.15–1.21] and multivariate-adjusted analyses (adjusted OR 1.24, 95% BCI 1.14–1.35) for each additional year of age. Nearly, 70.8% (*n=*34/48) were unaware of their status of living with HIV. Condom use with all sexual partners and condom-compatible-lubricant use with men were reported by 1.3% (95% CI 0.0–9.7).

**Conclusions:**

Although the epidemic in Swaziland is driven by high-risk heterosexual transmission, the burden of HIV and the HIV prevention, treatment and care needs of MSM have been understudied. The data presented here suggest that these men have specific HIV acquisition and transmission risks that differ from those of other reproductive-age adults. The scale-up in HIV services over the past decade has likely had limited benefit for MSM, potentially resulting in a scenario where epidemics of HIV among MSM expand in the context of slowing epidemics in the general population, a reality observed in most of the world.

## Introduction

Swaziland is a small, land-locked, lower-middle-income country that is surrounded by South Africa and Mozambique; it has a population of approximately 1.1 million people and a life expectancy at birth of approximately 48 years [1]. Similar to other Southern African countries, Swaziland has been severely affected by HIV, with over a quarter of its reproductive-age adults (15–49) estimated to be living with the virus, equating to an estimate of 170,000 people living with HIV [2]. Moreover, the incidence of HIV appears to have peaked in 1998–1999 at 4.6% [95% confidence interval (CI) 4.27–4.95], according to estimates by the Joint United Nations Programme on HIV/AIDS (UNAIDS), while in 2009 it was estimated to be 2.7% (95% CI 2.2–3.1%) [3–6]. There appear to have been further declines in incidence according to 6054 person-years of follow-up data from 18,154 people followed from December 2010 to June 2011 as part of the Swaziland HIV Incidence Measurement Survey (SHIMS) longitudinal cohort. Overall incidence was approximately 2.4% (95% CI 2.1–2.7%), with incidence estimated to be 3.1% (95% CI 2.6–3.7) among women as compared to 1.7% (95% CI 1.3–2.1) among men [7]. Indeed, women and girls have been more burdened with HIV than men throughout the history of the HIV epidemic in Swaziland, with the HIV prevalence among women 15–24 in 2006 being estimated to be 22.6% compared to 5.9% among age-matched men and boys [5].

The 2009 Swaziland Modes of Transmission study characterized major drivers of incident HIV infections to be multiple concurrent partnerships before and during marriage as well as low levels of male circumcision [8]. These risk factors were confirmed in the SHIMS study, with risk factors for incident HIV infections among both men and women including not being married or living alone, having higher numbers of sex partners and having serodiscordant or unknown HIV status partners [7]. There are no known HIV prevalence estimates for key populations in Swaziland, including female sex workers (FSW) or men who have sex with men (MSM) [9,10]. The 2009 Swazi Modes of Transmission Study indicates that both sex work and male–male sexual practices are reportedly infrequent and assumed to be minor drivers of HIV risks in the setting of a broadly generalized HIV epidemic. However, the prevalence of these risk factors has not been measured in the HIV surveillance systems that are used to inform the Modes of Transmission Surveys [11]. The last several years have witnessed an increase in the understanding of the potential vulnerabilities among these same key populations through targeted studies including MSM in neighbouring countries with similarly widespread HIV epidemics [12,13].

The largest body of data is available from South Africa, where the first study completed in 1983 of 250 MSM demonstrated a high prevalence of HIV, syphilis and hepatitis B virus [14]. More recently, a study of rural South African men found that approximately 3.6% of men studied (*n=*46) reported a history of having sex with another man [15]. Among these men, HIV prevalence was 3.6 times higher than among men not reporting male partners (95% CI 1.0–13.0, *p=*0.05) [16]. There have also been several targeted studies of MSM in urban centres across South Africa that consistently highlight a population of men who have specific risk factors for HIV acquisition and transmission and limited engagement in the continuum of HIV care [17–19]. Relatively recent studies from other countries, including Lesotho, Malawi, Namibia and Botswana, have shown similar diverse populations of MSM [16,20,21]. Diversity among populations of MSM across Southern Africa manifests through diverse sexual orientations and practices ranging from those who are gay identified, with primarily male sexual partners, to those who are straight identified, with both male and female sexual partners [22]. Diversity has also been measured in the range of HIV-related risk practices among MSM, including understanding of the HIV acquisition and transmission risks associated with unprotected anal intercourse and of the levels of use of condoms and condom-compatible lubricants (CCLs) [23].

To better characterize vulnerabilities and HIV prevention, treatment and care needs among MSM in Swaziland, a cross-sectional assessment was completed to provide an unbiased estimate of the prevalence of HIV and syphilis among adult MSM in Swaziland. This study was completed in equal collaboration with the Swaziland National AIDS Program (SNAP) in the Ministry of Health. This study further sought to describe the significant correlates of prevalent infections, including individual behavioural characteristics, and describe social and structural HIV-related factors and risks for HIV infection among MSM.

## Methods

### Sampling

MSM in Swaziland were recruited via respondent-driven sampling (RDS), a peer referral sampling method designed for data collection among hard-to-reach populations [24]. Potential participants were required to be at least 18 years of age, report anal sex with another man in the previous 12 months, be able to provide informed consent in either English or siSwati, be willing to undergo HIV and syphilis testing and possess a valid recruitment coupon.

### Survey administration and HIV testing

All participants completed face-to-face surveys and received HIV and syphilis tests on site. Surveys were administered by trained members of the research staff and lasted approximately one hour. The study was completely anonymous and did not collect any identifiable information; we used verbal rather than signed consent to further ensure anonymity. Questions on socio-demographics (e.g., age, marital status and education), behavioural HIV-related risk factors (e.g., HIV-related knowledge, attitudes and risk behaviours) and structural factors (e.g., stigma, discrimination and social cohesion) were included [25]. HIV and syphilis tests were conducted by trained phlebotomists or nurses, according to official Swazi guidelines. Test results, counselling and any necessary treatment (for syphilis) and/or referrals (for HIV) were provided on site. Participant surveys and test results were linked using reproducible, yet anonymous, 10-digit codes.

### Analytical methods

Population and individual weights were computed separately for each variable by the data-smoothing algorithm using RDS for Stata [26]. The weights were used to estimate RDS-adjusted univariate estimates with 95% bootstrapped confidence intervals (BCIs). Crude bivariate regression analyses were also conducted to assess the association of HIV status with demographic variables as well as a selection of variables either expected or shown to be associated with HIV status in the literature. All demographic variables were then included in the initial multivariate logistic regression model regardless of the estimated strength of their crude bivariate association with HIV status. Non-demographic variables were included in the initial multivariate model if the chi-square *p* value of association with HIV status was ≤0.25 in the bivariate analyses. Most of the demographics variables, however, dropped out of the final model after controlling for other independent variables.

Because regression analyses of RDS data using sample weights are complicated due to the fact that weights are variable-specific [27], RDS-adjusted bivariate and multivariate analyses were conducted using individualized weights that were specific to the outcome variable (i.e., HIV status) [27]. The adjusted odds ratio (aOR) estimates were not statistically different from the unadjusted estimates in the bivariate analyses, although some slight differences were observed in the multivariate analyses. Thus, only the unadjusted odds ratios (ORs) are reported for bivariate analyses, while both are presented in Table 1 for multivariate analyses. All data processing and analyses were conducted using Stata 12.1 [28].

### Missing data

Eleven out of the 324 participants were excluded from this analysis due to missing data on key RDS-related variables. There were 29 out of 313 participants with missing data on at least one variable used in the multivariate analyses. Only two variables had data missing for more than three participants: age at first sex with another man (*n* missing=4) and knowledge about the type of anal sex position that puts you most at risk of HIV infection (*n* missing=6). Two of the 29 participants with missing data were living with HIV; thus, the effective crude HIV prevalence used in the multivariate model was 17.6% (50/284) versus 16.6% (52/313) without missing data, and RDS-adjusted 13.4% (95% BCI: 7.9–19.7; homophily: HIV−=−0.0991, HIV+=0.134) versus 12.7% (BCI: 7.3–18.1; homophily: HIV−=−0.0899, HIV+=0.1358) Although the total number of cases with missing data is not very small (9.3%: 29/313), the number missing by variable is very small. Due to the small change in HIV prevalence in the analysis sample compared to the complete sample as shown in this article, no effort was made to impute missing data. The 29 cases were excluded in the multivariate regression models.

### Sample size calculation

The sample size was calculated based on the ability to detect significant differences in condom use among MSM living with HIV and those not living with HIV. There were no known estimates of condom use among MSM in Swaziland, but previous studies of MSM from nearby countries estimated that consistent condom use during anal sex with other men among MSM is approximately 50% [19]. In addition, a systematic review and meta-analysis of the literature on behavioural interventions targeting MSM have demonstrated that behavioural interventions can increase reported condom use by approximately 16.5% in all risk categories of MSM [29,30]. Thus, this study was powered on the assumption that those who have received information about preventing HIV infection from other men would have a 16.5% increase in reported consistent condom use. A power analysis demonstrated that with 80% power, we would require 160 participants. Estimates of appropriate design effects for RDS have varied in the literature, and we used a design effect of 2, planning for the accrual of 324 MSM [31]. This sample size facilitates the detection of significant differences in HIV-related protective practices, such as consistent condom use, and targeted HIV-prevention measures, and is sufficient for key social factors such as experiences with stigma and discrimination.

### Ethics

The study received approval for research on human participants from both the National Ethics Committee of Swaziland as well as the Institutional Review Board of the Johns Hopkins Bloomberg School of Public Health.

## Results

Three hundred and twenty-four men were accrued from six seeds over a range of between 1 and 14 waves of accrual, with the largest recruitment chain including 123 participants. As shown in Table 2, the majority of men sampled were under 30 years of age, with a mean age of 23.1 and a mode of 22. The crude sample was relatively educated, although highly educated men were oversampled in this study when comparing the crude results to RDS-adjusted results (unadjusted 23.0% and adjusted 14.8%). Most of the study participants had never been married (98.2%, 95% CI 96.5–99.9), with only 13 men reporting either cohabitating with a woman or being married to a woman. Similarly, only about one in 10 men reported having children (10.5%, 95% CI 5.8–15.3). Notably, the majority of the sample of participants did not self-identify as straight or heterosexual, with approximately two-thirds reporting being gay and one-third reporting being bisexual. When asked about gender identification, nearly a quarter of the sample reported identifying as a woman, although the adjusted proportion was 15.7% (95% CI 10.4–20.9). More than one-tenth of men reported having been to jail or prison (13.2%, 95% CI 7.9–18.6). Among 71 men aged 18–19, the HIV prevalence was 0%, compared to 8.8% (*n=*6/68) among participants aged 20–21, 15% (*n=*9/60) among participants aged 22–23, 21.4% (*n=*12/56) among participants aged 24–26, and finally 43.1% (*n=*25/58) among participants aged 27–43 (data not shown). In total, 29.2% (*n=*14/48) of participants living with HIV reported previously being told that they had HIV, although four participants not found to be living with HIV reported being given this diagnosis.

**Table 1:** Sociodemographic characteristics of a sample of men who have sex with men in Swaziland in 2011

Variable	Categories	*N*	Crude percentage	RDS-adjusted percentage	95% confidence interval		Homophily (−1 to +1)
Age in years	Under 21	94	30.0	36.3	27.4	45.2	0.199
	21–25	142	45.4	45.1	36.3	53.8	0.143
	26–30	56	17.9	12.0	7.2	16.7	0.148
	31 and older	21	6.7	6.7	2.9	10.4	0.026
	Some secondary, high school or lower	108	34.5	44.8	35.6	53.9	0.104
Education level	Completed secondary or high school	133	42.5	40.4	32.4	48.4	0.119
	Post-high-school vocational training or higher	72	23.0	14.8	9.4	20.2	0.180
	Unemployed	97	32.3	30.7	22.5	38.9	0.189
Employment status	Employed	101	33.7	27.5	19.5	35.5	0.203
	Student	102	34.0	41.8	32.6	51.0	−0.001
Marital status with a woman	Married or cohabitating	13	4.2	1.8	0.1	3.5	−0.018
	Single, never married	298	95.8	98.2	96.5	99.9	−1.423
Current housing tenure	Renting place	92	29.4	27.7	20.6	34.9	0.046
	Own place	51	16.3	18.3	12.0	24.6	−0.126
	Staying with someone	101	32.3	34.8	27.1	42.5	0.119
	Family	42	13.4	10.6	5.6	15.5	0.201
	Other	27	8.6	8.6	4.5	12.8	−0.095
Urban or rural origin	Urban	192	61.5	61.0	52.6	69.5	0.101
	Rural	120	38.5	39.0	30.5	47.4	0.172
Number of children	Zero	274	87.8	89.5	84.7	94.2	−0.174
	One or more	38	12.2	10.5	5.8	15.3	0.115
Gender Identification	Man	225	72.6	82.5	76.9	88.1	−0.29
	Woman	79	25.5	15.7	10.4	20.9	0.17
	Both	6	1.9	1.8	0.0	3.9	−0.018
Sexual orientation identification	Gay or homosexual	198	63.5	56.3	48.0	64.6	0.242
	Bisexual	109	34.9	40.5	32.3	48.6	0.062
	Heterosexual or straight	5	1.6	3.2	0.0	7.4	0.096
Age at first sex with a man	Under 21 years	238	77.0	77.6	70.7	84.6	0.110
	21 and above	71	23.0	22.4	15.4	29.3	0.083
Ever been to jail or prison?	No	276	88.2	86.8	81.4	92.1	0.216
	Yes	37	11.8	13.2	7.9	18.6	0.157

**Table 2:** HIV-related sexual and drug risk factors among MSM in Swaziland

Variable	Categories	*N*	Crude percentage	RDS-adjusted percentage	95% CI	
Number of male sexual partners in the past 12 months	1	103	33.0	42.1	34.0	50.2
	2	68	21.8	20.3	14.7	26.0
	3	70	22.4	20.3	14.2	26.5
	4+	71	22.8	17.2	11.7	22.7
Number of main male partners in the past 12 months	1	37	11.8	17.1	10.7	23.6
	2	183	58.5	57.2	49.7	64.7
	3	61	19.5	18.5	13.0	24.1
	4+	32	10.2	7.1	3.9	10.4
Number of male casual sexual partners in the past 12 months	None	132	42.4	46.6	39.0	54.2
	1–2	127	40.8	41.1	33.8	48.5
	3+	52	16.7	12.3	7.7	16.8
Number of female sexual partners in the past 12 months	None	198	64.3	53.6	44.9	62.4
	1	52	16.9	19.5	12.5	26.5
	2	29	9.4	15.1	8.7	21.6
	3+	29	9.4	11.8	6.2	17.4
Number of both male and female sex partners in the past 12 months	Only male	221	70.6	64.3	56.4	72.3
	Male and female	92	29.4	35.7	27.7	43.6
In general, how often have you used a condom in the past six months?	Never or almost never	30	9.7	11.5	6.0	17.0
	Sometimes	79	25.6	27.0	19.7	34.4
	Almost always	58	18.8	18.9	12.9	24.9
	Always	141	45.8	42.5	34.7	50.3
Had unprotected insertive anal sex in the past 12 months	No	190	60.9	55.5	46.8	64.2
	Yes	122	39.1	44.5	35.8	53.2
Had unprotected receptive anal sex in the past 12 months	No	211	68.7	69.8	62.7	76.8
	Yes	96	31.3	30.2	23.2	37.3
Condom use with main male partners in the past 12 months	Not always	137	47.2	51.9	41.8	62.0
	Always	153	52.8	48.1	38.0	58.2
Condom use with casual male partners in the past 12 months	Not always	54	17.3	16.6	10.2	23.0
	Always	150	47.9	46.1	38.6	53.6
	No casual partner	109	34.8	37.3	29.8	44.9
Condom use with regular female partners in the past 12 months	Not always	47	49.5	61.8	41.3	82.3
	Always	48	50.5	38.2	17.7	58.7
Condom use with casual female partners in the past 12 months	Not always	31	45.6	55.6	25.2	86.0
	Always	37	54.4	44.4	14.0	74.8
Used water-based lubricant (WBL) in the past 12 months	No	203	64.9	76.3	69.8	82.8
	Uses WBL	110	35.1	23.7	17.2	30.2
Safe sex with men (condoms and water-based lubricant) in the past 12 months	Does not	257	82.1	87.4	82.4	92.4
	Does	56	17.9	12.6	7.6	17.6
Safe sex with women (condoms) in the past 12 months	Does not	66	60.0	75.3	60.5	90.0
	Does	44	40.0	24.7	10.0	39.5
Safe sex with both men and women in the past 12 months	Does not	104	95.7	98.7	90.3	100.0
	Does	6	4.3	1.3	0.0	9.7
Injected illicit drugs in the past 12 months	No	304	97.1	97.7	96.1	99.3
	Yes	9	2.9	2.3	0.7	3.9
Used non-injection illicit drugs in the past 12 months	No	203	65.1	66.4	58.5	74.3
	Yes	109	34.9	33.6	25.7	41.5
Used alcohol in the last month	None	121	39.0	36.1	28.7	43.5
	At least one day	189	61.0	63.9	56.5	71.3
Which is the safest lubricant to use during anal sex?	Non-WBL	128	49.8	63.5	53.6	73.4
	WBL	129	50.2	36.5	26.6	46.4
Can you get HIV from sharing a needle to inject illegal drugs?	No	3	1.0	1.0	−0.2	2.2
	Yes	303	99.0	99.0	97.8	100.2
What type of sex puts you most at risk for HIV infection?	Vaginal	110	35.1	43.7	36.1	51.2
	Anal	75	24.0	18.2	12.4	24.0
	Oral	25	8.0	8.6	3.8	13.5
Which type of anal sex position puts you most at risk for HIV infection?	Insertive (top)	63	20.5	24.0	17.1	30.9
	Receptive (bottom)	95	30.9	31.1	23.8	38.4
	Insertive and receptive anal sex carry equal risk	149	48.5	44.9	36.6	53.2
Answered all of the above correctly	No	278	88.8	90.9	87.0	94.8
	Yes	35	11.2	9.1	5.2	13.0

The majority of men had multiple male sexual partners over the past 12 months (57.9%, 95% CI 49.8–66.0). Moreover, most study participants had multiple main sexual partners, or boyfriends, over the past 12 months (82.9%, 95% CI 76.4–89.3) (Table 3). About one-third of participants reported having had both male and female sexual partners in the previous 12 months (35.7%, 95% CI 27.7–43.6). Approximately one-half of the participants reported always using condoms during sex, although significant numbers of men reported both unprotected insertive and receptive anal intercourse in the past 12 months. Condom use was not significantly different between main and casual male or female partners. Overall, safe sex with other men, defined as always using condoms and water-based lubricants over the last 12 months, was not common, with 12.6% (95% CI 7.6–12.6) measured to report this behaviour. Safe sex, defined as condom use with all sexual partners over the last 12 months, was significantly higher with female partners (at 40.0% in the crude assessment) than with male partners (*p<*0.05). Overall, safe sex with all sexual partners was uncommon and was reported by 4.3% (RDS-adjusted 1.3%, 95% CI 0.0–9.7). Knowledge of basic questions related to safe sex for MSM, including sexual positioning, type of sexual act and lubricant use, was low, with 11.2% (RDS-adjusted 9.1%, 95% CI 5.2–13.0) of participants providing correct answers.

**Table 3:** Service uptake and structural HIV risks among MSM in Swaziland

Variable	Categories	*N*	Crude percentage	RDS-adjusted percentage	95% CI	
Participated in any meetings related to HIV/AIDS in the past 12 months	No	175	55.9	58.5	51.1	65.8
	Yes	138	44.1	41.5	34.2	48.9
Participated in any meetings related to HIV/AIDS in the past 12 months related to MSM	No	243	78.4	83.5	78.1	88.8
	Yes	67	21.6	16.5	11.2	21.9
Received information about preventing HIV from sex with women in last 12 months	No	60	19.4	20.9	14.5	27.2
	Yes	250	80.6	79.1	72.8	85.5
Received information about preventing HIV from sex with other men in last 12 months	No	226	72.4	78.5	72.9	84.1
	Yes	86	27.6	21.5	15.9	27.1
Level of concern related to HIV in the last 12 months	Not worried	86	27.6	31.8	24.9	38.8
	Not very worried	61	19.6	18.2	12.1	24.2
	Somewhat worried	52	16.7	16.8	10.0	23.6
	Very worried	113	36.2	33.2	26.0	40.3
Access to condoms: do you have them when you need them?	No access	3	1.0	1.0	-0.4	2.3
	Difficult or little access	58	18.6	16.8	11.2	22.4
	Some access	36	11.6	12.6	7.2	18.0
	Very easy access	214	68.8	69.6	61.9	77.4
Symptoms of sexually transmitted infection (STI) in the past 12 months	No	247	79.2	78.5	72.4	84.6
	Yes	65	20.8	21.5	15.4	27.6
Tested for STI in the past 12 months	No	266	87.2	86.1	80.5	91.7
	Yes	39	12.8	13.9	8.3	19.5
Diagnosis of STI in the past 12 months	No	287	92.6	92.2	88.3	96.1
	Yes	23	7.4	7.8	3.9	11.7
Been tested for HIV in the past 12 months	No	144	46.0	49.3	41.8	56.8
	Yes, once	94	30.0	31.2	24.2	38.2
	Yes, >1	75	24.0	19.5	13.5	25.4
Ever been told that you have HIV?	No	284	94.0	95.7	92.5	98.9
	Yes	18	6.0	4.3	1.1	7.5
Perceived human rights violations	No	63	20.1	20.4	14.5	26.3
	Yes	250	79.9	79.6	73.7	85.5
Experienced human rights violations	No	152	48.6	48.9	40.5	57.2
	Yes	161	51.4	51.1	42.8	59.5
Disclosure to healthcare workers	No	218	69.6	75.0	69.0	81.0
	Yes	95	30.4	25.0	19.0	31.0
Disclosure to family	No	146	46.6	56.0	48.3	63.6
	Yes	167	53.4	44.0	36.4	51.7

Table 4 demonstrates levels of service uptake, with evidence of statistically significantly lower levels of access to targeted services focused on preventing HIV transmission via sex between men as compared to sex between men and women (*p<*0.05 for both). Notably, only about half of the sample was somewhat or very worried about HIV. Just under half of the men who had symptoms of a sexually transmitted infection (STI) were tested in the previous 12 months, with 7.8% (95% CI 3.9–11.7) diagnosed in this same time frame. About half of the sample had been tested for HIV in the previous 12 months (50.7%, 95% CI 43.2–59.2), including some who were tested more than one time. Reports of any experienced rights violations related to sexual practices, including denial of care, police-mediated violence and physical or verbal harassment, were reported by about half of the sample, although perceived rights violations related to sexual orientation (fear of seeking healthcare and fear of walking in the community) were more common, with 79.6% (95% CI 73.7–85.5) calculated to report this. Disclosure of sexual practices to healthcare workers was reported by one-quarter of the sample (25.0%, 95% CI 19.0–31.0), whereas about half of the participants (44.0%, 95% CI 36.4–51.7) had reported disclosure of sexual practices to a family member.

**Table 4:** Bivariate and multivariate associations with HIV status among men who have sex with men (MSM) in Swaziland

		Bivariate		Multivariate – crude		Multivariate – RDS weighted	
				
Variable	Categories	Estimate	[95% CI]	Estimate	[95% CI]	Weighted estimate	Weighted estimate 95% CI
Current age	Years	1.23	[1.15–1.31]	1.24***	[1.14–1.35]	1.28***	[1.15–1.43]
	Man	1		1		1	
Gender	Woman	2.14	[0.90–5.05]	3.96**	[1.66–9.43]	3.23*	[1.07–9.71]
	Both	–		–		–	
Education level	Some secondary, high school or lower	1		1		1	
	Completed secondary or high school	1.06	[0.44–2.56]	1.32	[0.54–3.18]	1.51	[0.46–5.00]
	Post-high-school vocational training or higher	1.34	[0.47–3.77]	0.56	[0.20–1.57]	0.62	[0.18–2.16]
Age at first sex with another man	Under 21 years	1		1		1	
	21 and above	2.38	[0.99–5.72]	1.24	[0.49–3.14]	0.71	[0.18–2.75]
Urban or rural origin	Urban	1		1		1	
	Rural	1.99	[0.91–4.35]	0.79	[0.34–1.79]	1.33	[0.45–3.93]
Ever been to jail or prison?	No	1		1		1	
	Yes	2.75*	[1.08–7.00]	3.00*	[1.01–8.85]	4.37*	[1.38–13.84]
Diagnosis with an STI other than HIV in last 12 months	No	1		1		1	
	Yes	1.57	[0.49–5.07]	6.26**	[1.68–23.39]	4.30*	[1.04–17.72]
Number of casual male partners in the last 12 months	None	1		1		1	
	1–2	0.51	[0.20–1.26]	0.33*	[0.13–0.85]	0.26*	[0.08–0.85]
	3+	1.12	[0.42–2.98]	1.04	[0.37–2.95]	0.50	[0.13–1.97]
Which type of anal sex position puts you most at risk for HIV infection?	Insertive (top)	1		1		1	
	Receptive (bottom)	0.91	[0.33–2.54]	0.49	[0.17–1.42]	0.53	[0.14–2.08]
	Insertive and receptive anal sex carry equal risk	0.96	[0.37–2.54]	0.39	[0.14–1.06]	1.43	[0.32–6.41]
In the past 12 months, have you used any non-injectable drug that was not prescribed?	No	1		1		1	
	Yes	0.84	[0.375–1.865]	0.356*	[0.136–0.935]	0.366	[0.12–1.11]
What kind of access to condoms do you have when you need them?	No access	1		1		1	
	Difficult or little access	0.13	[0.007–2.380]	0.008**	[0.000–0.224]	0.031	[0.001–1.23]
	Some access	0.36	[0.021–6.115]	0.043	[0.002–1.022]	0.170	[0.01–5.35]
	Very easy access	0.49	[0.034–7.054]	0.043*	[0.002–0.893]	0.264	[0.007–10.020]
In the past 30 days, how many days did you drink at least one drink of alcohol?	Zero	1		1		1	
	At least one day	1.30	[0.55–3.07]	1.81	[0.74–4.41]	2.19	[0.60–7.96]
Analysis sample						284	284

Exponentiated coefficients; 95% CI=95% confidence intervals

* *p*<0.05

** *p*<0.01

*** *p*<0.001.

HIV prevalence was strongly correlated with age in both bivariate analyses (OR 1.23, 95% BCI 1.15–1.21) for each year of age and multivariate-adjusted analyses (aOR 1.24, 95% BCI 1.14–1.35) (Table 1). Other statistically significant associations with HIV in adjusted analyses included identifying as the female gender, having ever been to jail or prison, having lower numbers of casual partners, being diagnosed with an STI in the last 12 months and having easier access to condoms.

## Discussion

**Figure 1:**
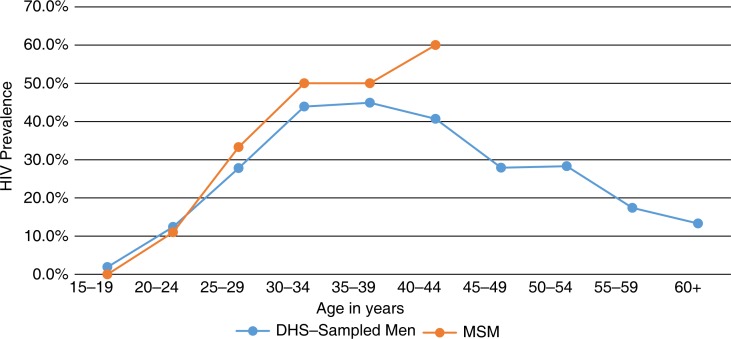
Prevalence of HIV by age among Swazi men who have sex with men, 2011.

In the country with the highest HIV prevalence in the world, this study describes the burden of HIV and associated characteristics among MSM who were accrued using RDS. Interpreting the prevalence of HIV among MSM and its relationship with the widespread and generalized female-predominant epidemic in Swaziland is challenging on a number of levels. The significant association between HIV and age suggests that the expanding epidemic among MSM in Swaziland is not new and represents cumulative HIV acquisition risk exposures. The burden of HIV among all men aged 15–19 is approximately 2% in Swaziland, increasing to 12.4% among those aged 20–24 and up to 44.9% among those aged 35–39. While the participants in our study were relatively young, the HIV prevalence was consistent with that of general reproductive-age men until age 24–26, when the prevalence of HIV among age-matched MSM appears to be higher than that of other men sampled as part of the Swazi DHS study (Figure 1) [2]. Given that relatively few men in our sample reported female sexual partners, their HIV acquisition and transmission risks are likely different from those of other men in Swaziland and potentially more related to anal intercourse. Conversely, Swaziland may be among a small number of countries where even the low acquisition risks associated with insertive penile-vaginal intercourse is counterbalanced by the significantly higher HIV prevalence among women, resulting in significant acquisition risks associated with sex with women. However, the idea that acquisition risk for MSM primarily related to sex with other men is reinforced by the results that condom use was lower with male sexual partners than with female sexual partners. Condoms being used more frequently during sex with women as compared to sex with other men have been observed in other studies of MSM across Sub-Saharan Africa and provide an argument against MSM being a population that bridges the HIV epidemic from within their sexual networks to lower risk heterosexual networks [19,20,32,33]. However, to answer this question, phylogenetic studies and the characterization of sexual networks are needed to better describe patterns of HIV transmission.

Participants were far more likely to have received information about preventing HIV infection during sex with women as compared to sex with other men. This lack of access to or uptake of information, education and communication services has resulted in participants in this study having a limited knowledge base of the sexual risks associated with same-sex practices. Primarily, participants incorrectly believed that unprotected penile-vaginal intercourse was associated with the highest risk of HIV transmission, consistent with earlier studies of MSM across Sub-Saharan Africa. Numerous studies have shown the opposite: HIV is far more efficiently transmitted during anal intercourse as compared to vaginal intercourse [13,34]. There was also limited knowledge related to the importance of water-based lubricants being CCLs, which is especially important during anal intercourse given the absence of physiological lubrication in the anal canal. The importance of CCL was underscored as ultimately being the determining factor in just six study participants reporting safe sex with all partners in this study. Thus, while there is significant provision of general HIV-prevention messaging across Swaziland, there has been limited information focused on educating MSM on how to prevent HIV acquisition and transmission during sex with other men. Data suggest that starting with simple and proven approaches, including peer education programmes, is necessary to educate these men about their risks and protective behavioural strategies [35]. However, these approaches will likely not be sufficient to change the trajectory of HIV epidemics given the high risk of infection associated with unprotected anal intercourse with non-virally suppressed HIV serodiscordant partners. Thus, moving forward necessitates assessing the feasibility of combination approaches that integrate advances such as antiretroviral-mediated pre-exposure prophylaxis and universal access to antiretroviral therapy for people living with HIV [13]. However, the success or failure in achieving coverage with these HIV prevention, treatment and care approaches among MSM will, in part, be determined by the level of stigma affecting MSM.

It is now broadly accepted that addressing the needs of people living with HIV is vital to protect their own health as well as prevent onward transmission of HIV [36]. In addition, mean and total viral loads in a population have been linked to population-level transmission rates of HIV [37]. Only a quarter of the men living with HIV in this study were aware of their diagnosis, demonstrating the need to increase HIV testing, linkage to CD4 testing, and antiretroviral treatment and adherence support for those who are eligible. A recent systematic review and meta-analysis of self-testing for HIV in both low- and high-risk populations demonstrated that self-testing was both appropriate and associated with increased uptake of HIV tests [38]. This may be especially relevant in the Swazi context, where fear of seeking healthcare was prevalent, suggesting the need to study new strategies to overcome barriers to HIV testing among MSM in Swaziland, including leveraging community networks and potentially self-testing [39]. In this study, being a person living with HIV was associated with lower numbers of casual male partners in the last 12 months. This relationship appeared to be stronger among those who were aware of their status, although it was not statistically significant because of limited numbers. In addition, these data are consistent with earlier research findings that simply being made aware of one's status of living with HIV can change one's sexual practices to decrease onward transmission [40]. This further argues for implementation science research focused on optimal strategies to scale-up HIV testing for MSM in Swaziland [41].

Over one-quarter of participants in this study self-identified as women, and this was independently associated with living with HIV. There is nearly a complete dearth of information related to HIV among transgender people across Sub-Saharan Africa [42,43]. However, where transgender people have been studied, they have been found to be the most vulnerable to HIV acquisition because of increased structural barriers to HIV prevention, treatment and care services and because of increased sexual risks, including unprotected receptive anal intercourse [43]. Given the limited information available about transgender people, transgender was assessed in this study as both a sexual orientation and a gender identity. There was a significant disconnect between these two as no participants self-identified as being transgender. Ultimately, further ethnographic research is needed to better understand the HIV-prevention needs of transgender people in Swaziland.

Having been to jail was also independently associated with living with HIV among MSM in this study. Globally, incarceration has been shown to be an important risk factor for HIV, given the limited access to HIV-prevention services such as condoms and CCLs, the interruption of HIV treatment as well as exposure to higher risk sexual partners [44–47]. While further research is needed on same-sex practices within jails, there is likely a need to provide HIV-prevention services for men in Swazi prison settings [47].

The methods employed in this study have several limitations. While RDS is an effective approach to characterize asymptotically unbiased estimates intended to approximate population-based estimates of characteristics in the absence of a meaningful sampling frame, there are still several uncertainties in the most appropriate tools for interpretation of these data [48]. Moreover, the sample of men accrued here was relatively young, consistent with recruitment challenges observed in other studies of MSM across sub-Saharan Africa. While we conducted significant engagement with older MSM, fear associated with inadvertent disclosure limited their participation in the study. Only with improved social environments will more information about the needs of older MSM become available in difficult contexts [49]. In addition, while RDS was used to accrue a diverse sample, all of the seeds were connected with Rock of Hope, a newly registered organization serving the needs of lesbian, gay, bisexual and transgender populations in Swaziland. We thus may have overestimated actual service uptake among MSM in Swaziland.

## Conclusions

The implementation of the research project was guided by recent guidelines to inform HIV-related research with MSM in rights-constrained environments [50]. While these men had not been previously engaged in research on HIV prevention, treatment and care, the success of this study highlights the fact that accrual of this population is both feasible and informative for the HIV response in Swaziland. Moreover, the interconnected social and sexual networks leveraged for accrual can likely serve to disseminate HIV-prevention approaches via MSM throughout the country. While the epidemic in Swaziland is one driven by heterosexual transmission, the burden of HIV and the HIV prevention, treatment and care needs of MSM have been understudied, and these men have been underserved in the context of large-scale programmes [51]. The data presented here suggest that these men have specific HIV acquisition and transmission risks that differ from those of other reproductive-age adults. Encouragingly, Swaziland has seen declines in the rate of new HIV infections over the last seven years, and these declines are related to HIV testing and treatment scale-up [5]. However, the increase in HIV services likely has had limited benefit for MSM, which may result in a scenario where epidemics of MSM expand in the context of slowing epidemics in the general population – a reality observed in most of the world [13].
